# The dynamics of food shopping behavior: Exploring travel patterns in low-income Detroit neighborhoods experiencing extreme disinvestment using agent-based modeling

**DOI:** 10.1371/journal.pone.0243501

**Published:** 2020-12-21

**Authors:** Igor Vojnovic, Arika Ligmann-Zielinska, Timothy F. LeDoux

**Affiliations:** 1 Global Urban Studies Program / Department of Geography, Environment, and Spatial Sciences / School of Planning, Design and Construction, Michigan State University, East Lansing, Michigan, United States of America; 2 Department of Geography, Environment, and Spatial Sciences, Michigan State University, East Lansing, Michigan, United States of America; 3 Geography, Planning and Sustainability, Westfield State University, Westfield, Massachusetts, United States of America; Peking University Shenzhen Graduate School, CHINA

## Abstract

Only a handful of studies have leveraged agent-based models (ABMs) to examine public health outcomes and policy interventions associated with uneven urban food environments. While providing keen insights about the role of ABMs in studying urban food environments, these studies underutilize real-world data on individual behavior in their models. This study provides a unique contribution to the ABM and food access literature by utilizing survey data to develop an empirically-rich spatially-explicit ABM of food access. This model is used to simulate and scrutinize individual travel behavior associated with accessing food in low-income neighborhoods experiencing disinvestment in Detroit (Michigan), U.S. In particular, the relationship between trip frequencies, mode of travel, store choice, and distances traveled among individuals grouped into strata based on selected sociodemographic characteristics, including household income and age, is examined. Results reveal a diversified picture of not only how income and age shape food shopping travel but also the different thresholds of tolerance for non-motorized travel to stores. Younger and poorer population subgroups have a higher propensity to utilize non-motorized travel for shopping than older and wealthier subgroups. While all groups tend to travel considerable distances outside their immediate local food environment, different sociodemographic groups maintain unique spatial patterns of grocery-shopping behavior throughout the city and the suburbs. Overall, these results challenge foundational tenets in urban planning and design, regarding the specific characteristics necessary in the built environment to facilitate accessibility to urban amenities, such as grocery stores. In neighborhoods experiencing disinvestment, sociodemographic conditions play a more important role than the built environment in shaping food accessibility and ultimately travel behavior.

## Introduction

Throughout the second half of the 20^th^ century, the coupling of rapid suburbanization and disinvestment within urban cores has reshaped the spatial structure of America’s cities. A consequence was the deterioration of accessibility for large urban population segments to reaching basic daily amenities [1–4]. With the suburbanization of residents, businesses, and the tax base more broadly, urban commercial destinations–from personal and health services to healthy restaurant options and retail outlets–have been steadily moving to locations in the more distant metropolitan peripheries. Within this context, considerable research interest has been placed on examining the availability, accessibility, and quality of healthy food options within urban neighborhoods experiencing disinvestment and decline. Particular vulnerabilities have been recognized among poor, minority populations living within cities, who are faced with limited access to culturally appropriate and nutritious food within their neighborhoods. Due to their low incomes, these population subgroups also tend to have more restricted mobility [5–10]. Their travel constraints are not only shaped by the limited private automobile ownership among lower-income households, but also due to the more inadequate public transit access in cities experiencing disinvestment and decline.

Considerable criticism, and from different disciplinary perspectives, have been raised regarding the nature of post-World War II development processes encouraging the coupling of excessive suburbanization and urban decline across U.S. cities. These critiques have been leveled not only at the resulting social, economic and environmental impacts of these development patterns but also, the growing health dimensions, including unhealthy neighborhood food environments that have emerged as a result of this evolving urban spatial structure [11–13]. In the 21^st^ century, there has been a growing concern with the more limited healthy food options that are available in marginalized urban neighborhoods. As a result, inadequate dietary intake and related adverse health outcomes, including obesity, poorer mental health, cancer, cardiovascular disease, and diabetes, are omnipresent [14, 15]. These have been accentuated with the recent COVID-19 outbreak. Urban spatial patterns, stemming from the coupling of extreme suburbanization and the excessive urban disinvestment and decline, are perhaps nowhere as evident as across the Detroit region (Michigan).

Recently there has been a significant increase in utilizing complex systems modeling in public health and epidemiology [16–19], with a special focus on agent-based models (ABMs) in analyzing diet and physical activity. Applications include urban walkability [20–23], diet and obesity [24, 25], and food access [26]. There are multiple rationales behind adopting the agent-based paradigm to study the health implications of food access constrained by both physical space and social status. For example, Widener et al. [27] and Abel and Faust [28] demonstrate the efficacy of ABMs for testing specific policy interventions in food deserts: introducing new grocery stores, expanding public transit options, changing the food stock in selected stores (with more focus on fresh produce), or proposing mobile market distribution systems. Both studies utilize geographical data to emulate real-world systems, in Buffalo (New York) and Austin (Texas), U.S. What sets these examples apart is that they provide state-of-the-art quantitative tools that allow for measurable and transparent assessment of costly interventions.

Another article of note is by Koh et al. [29], who report on an ABM that emulates the urban food environment in Columbus (Ohio), U.S. Household agents, equipped with selected sociodemographic characteristics, shop in supermarkets based on empirically derived probabilities. The major output of the model is food availability per household, measured using a monthly food availability index. The model is empirically rich, built on sociodemographic data, surveys, and vector GIS. Food availability is predominantly driven by income and car ownership. Unlike the model presented below, the ABM by Koh et al. does not directly use a road network for food travel, and operates on a premise that agents select the nearest stores. In contrast, agents in our model select stores from a pool identified by respondents, and visit them based on empirically-derived frequencies. Finally, the objective of the model presented here is different–we focus on travel distance versus mode of transportation, rather than food availability and dietary intake patterns.

Block et al. [26] report on another interesting model aimed at urban food market analysis. Their objective is to simulate food consumption in Eindhoven, Netherlands. Household agents are characterized by household composition, income, and food preferences. The model does not use vector data, which mimics urban environments more realistically, and store selection is based on calculated utility that combines different factors affecting the purchase of nutritious food. Just like the ABM by Koh et al., the model does not directly use road network traversal and store selection is not driven by a pool of preferred stores obtained through a survey.

While these examples are undoubtedly novel and provide a unique basis for a conversation about the role of modeling in studying complex urban systems, they also have limitations, one of which is the underutilization of real-world data on individual decision-making. Through the example reported herein, we want to demonstrate the role of habitual behavior in grocery shopping. We argue that, for the proposed policies to be successful, researchers need to investigate how individual households make their store selection and resulting dietary decisions. A comprehensive exploration of grocery shopping habits will allow for designing policies that are not only geared towards specific geographic communities [28], but also sociodemographic groups.

This paper reports on the ongoing development of an empirically-rich, spatially-explicit ABM of food access (called fABM, 'f' for food) and applying it in the Detroit context. We are interested in simulating the relationship between the mode of travel, and the distance traveled to buy groceries by different sociodemographic groups. In particular, we want to evaluate how the choice of mode changes with distance to the selected store. Using complex urban systems modeling, two key travel behavior and food access questions will be answered, both of which focus on exploring food shopping and adaptation patterns of low-income residents living in Detroit neighborhoods experiencing extreme disinvestment and decline. The first question examines how disadvantaged consumers spatially adapt their food-shopping travel when no major national/regional supermarket chains are found within the city boundaries? The second question examines how shopping travel patterns of residents living in neighborhoods experiencing disinvestment and decline differ by income and age? Our model is highly empirically-grounded–it was almost entirely developed from data collected through a household survey [30, 31]. It allowed us to group the population into strata based on selected sociodemographics. We discovered that these groups have unique patterns of grocery-shopping behavior. The number of weekly trips, the choice of stores, and the use of different modes of transportation–all paint a diversified and complex picture of how individual families purchase food.

The rest of the paper is organized as follows. In the Case Study, Materials, Data, and Methods section, we describe the research area, data collected, and fABM development and experimentation. What follows are Results, which summarize the significant observations from the simulations. In the Discussion section, we examine the significance of the research results, including from an urban planning and design perspective, and we conclude the paper by exploring the significance of this research, including to agent-based models.

## Case study, materials, data, and methods

The City of Detroit’s population reached a high of about 1.85 million in 1950 [32]. Six decades later, by the 2010 Census, Detroit’s population would decline to 713,777 people, with the current number of inhabitants estimated at around 670,031 [33, 34]. Just between 2000 and 2010, the city’s population decreased from 951,270 to 713,777, as some 24,000 people abandoned the city every year, about 63 people per day [33, 35, 36]. The city was built to accommodate 1.85 million people, but with only some 670,000 people currently residing within its boundaries, large sections of Detroit remain vacant.

The urban decline in Detroit resembles many of the older, northern rustbelt cities, including Buffalo (New York), Cleveland (Ohio), and Flint (Michigan). Into the 21^st^ century, however, perhaps the model of the coupling between rapid urban disinvestment and ongoing suburbanization continues to be evident across the Detroit region. The impact of this decline can also be placed into a monetary context, the city’s fiscal capacity as evident with the local assessed value of property. Over a six-decade period, ending in 2012, the total property value in the City of Detroit declined from $37 billion to just $9.4 billion (in 2012 dollars) [37]. A similar pattern of decline is also evident in the wholesale trade in grocery and related products. While the City of Detroit had 629 businesses, doing more than $11.8 billion (2012 dollars) in sales in 1967, the figure dropped to 101 establishments with sales of about $2.9 billion (2012 dollars) just four decades later [32, 35].

**Fig 1:**
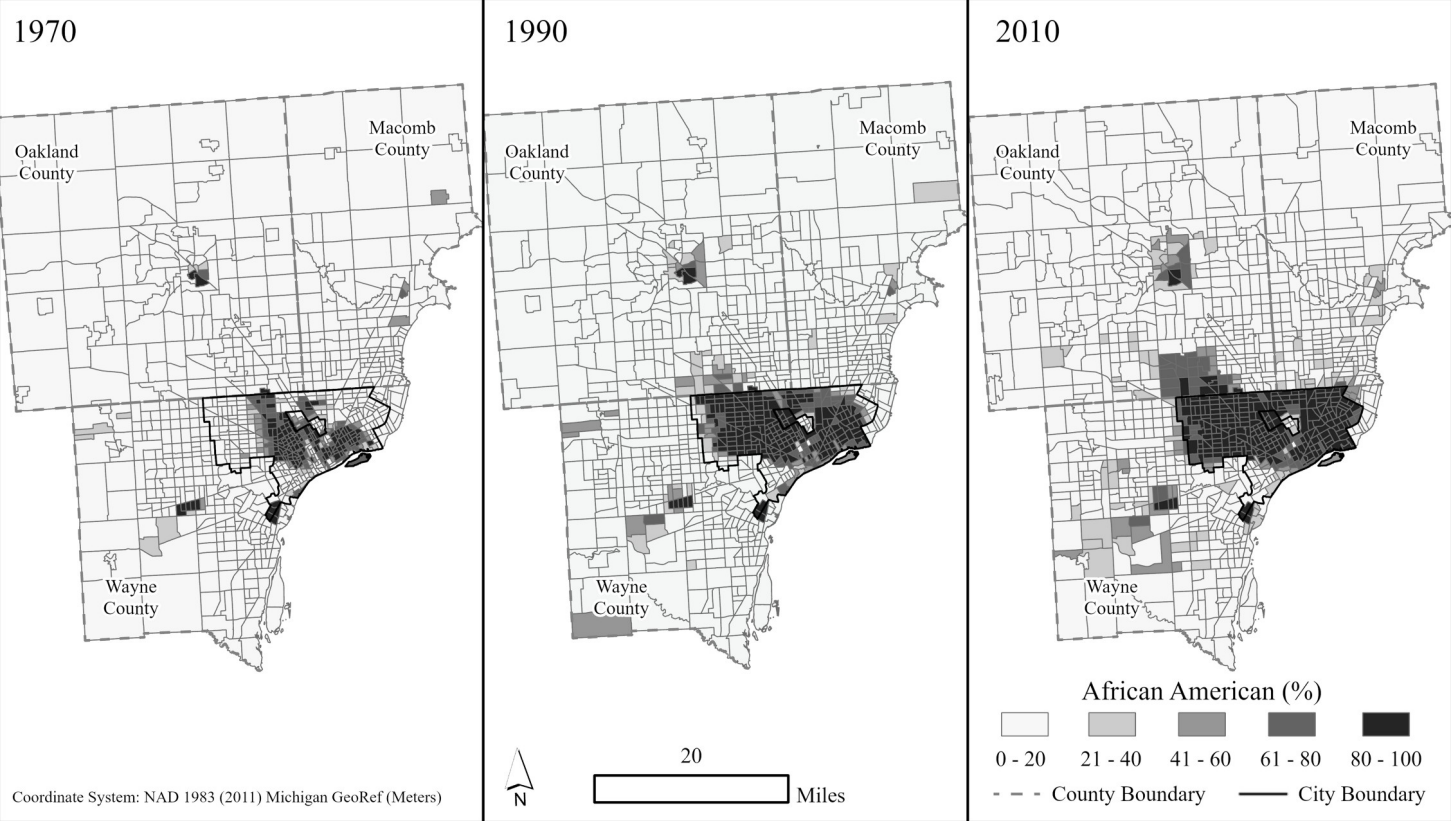
The blackening of Detroit (1970, 1990, and 2010). Map generated by authors, using public domain data from [38, 39].

Across the Detroit region, there has also been a clear class and racial dimension to the suburbanization, the classic post-World War II U.S. experience of *white flight* (Fig 1). In 2010, the City of Detroit’s population was 83% black, a figure significantly up from the city’s 16% black composition in 1950 [32, 33]. This stands in contrast with the Detroit Metro suburbs, where, in 2010, over 97% of all whites resided, while less than 3% of the white Metro population lived within the City of Detroit [33].

**Fig 2:**
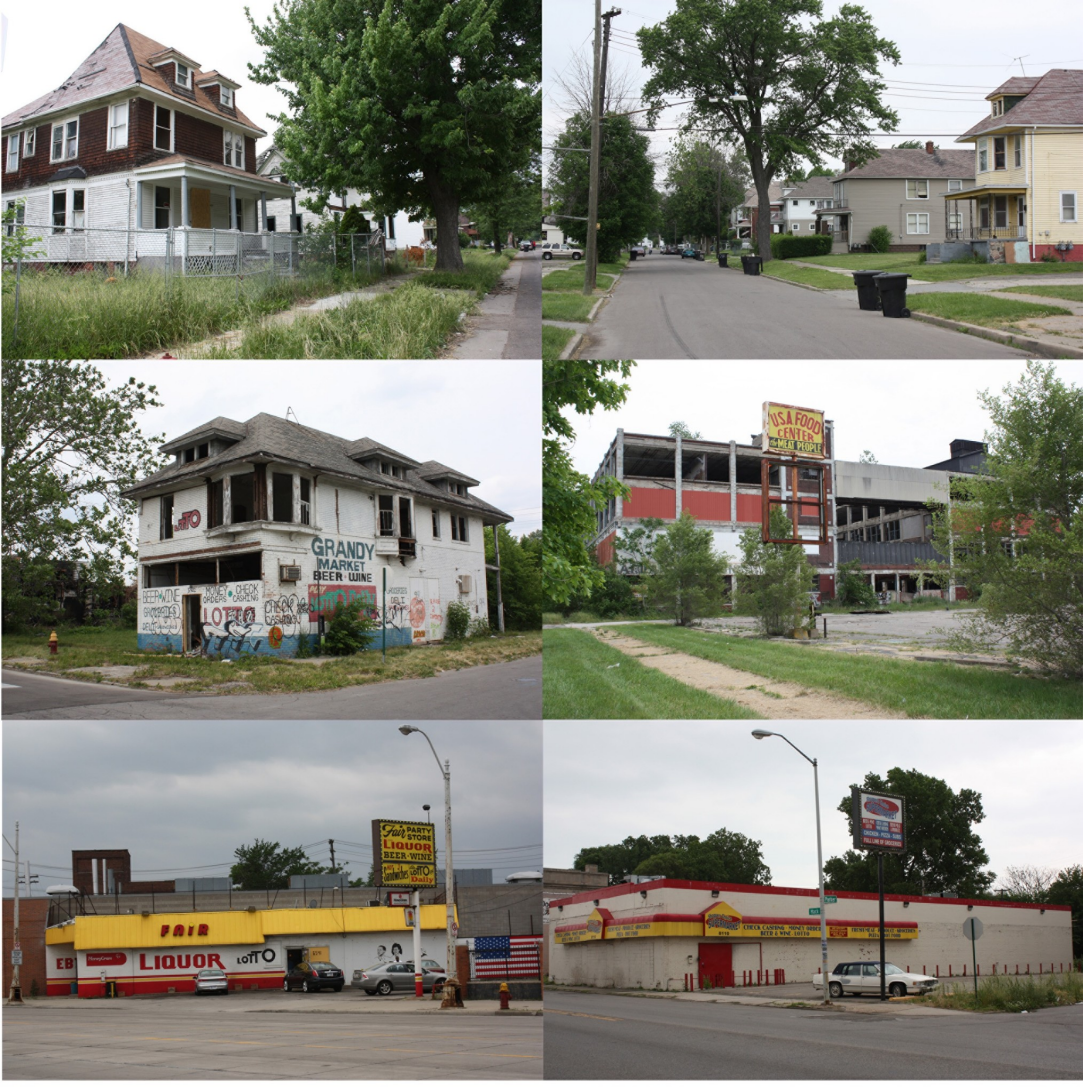
Urban decline in the lower east side Detroit neighborhoods, with images showing the neighborhoods, residences, and vacated and open stores.

There is also a class imprint paralleling this racial segregation across the Detroit region. Between 2014 and 2018, the poverty rate in the City of Detroit averaged 36.4%, see Fig 2. In contrast, it averaged only 8.6% in its wealthy suburbs of Oakland County, with the above figures reflecting the regional coupling of *white flight* and urban disinvestment [40]. It is within this context of urban disinvestment and sparse food environments that our study examines food access and travel for grocery shopping among different population subgroups living in highly segregated Detroit’s lower east side neighborhoods, which are primarily composed of African American, lower-income residents.

**Fig 3:**
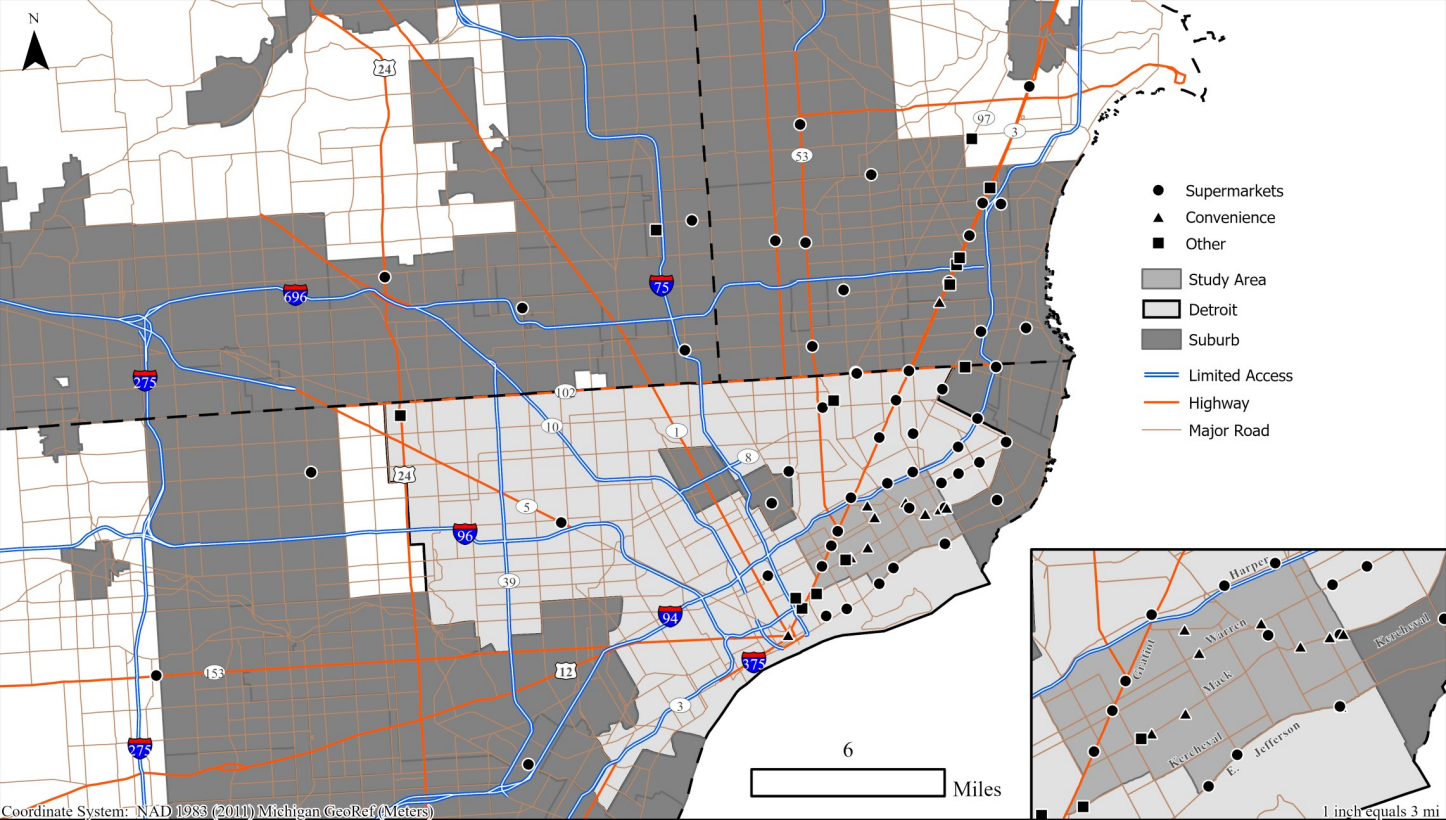
The store types and the three shopping zones*–* 1) the case study neighborhoods, 2) the City of Detroit, and 3) the Detroit suburbs. Only stores identified by survey respondents are used in this study (as shown in the map). Map generated by authors, using public domain data from [39] under a CC BY license.

The case study neighborhoods consist of 23 census tracts, with approximately 14,700 households, located in Detroit’s lower east side (Fig 3). The population is primarily African-American (approximately 94%), low-income (annual median household income of $20,822), with over 30% of households lacking a vehicle. These are older Detroit neighborhoods, with a built environment that can best be described as mid-density, high connectivity, and mixed land use (integrated residential, commercial, public, and industrial uses). The vast majority of houses are single-family. Many of the homes within these neighborhoods have been vacated, with noticeable increases in abandonment starting with the Great Recession, 2007.

A stratified random 8-page mail survey was sent-out and collected over the second-half of 2008 and into early-2009. Michigan State University (IRB# X06-258) approval was granted to collect these data once written consent was obtained. The survey collected data on typical weekly travel, with consideration given to seasonal distinctions. The travel data collected included information on destinations, frequency, and mode of travel, and it involved a full array of trips, including to work, shopping, restaurants, personal services, and leisure. Information was also collected on personal variables (sociodemographics), diet, and physical activity. Respondents had to be 18 years or older to complete the survey, and they received a gift card to a local supermarket once the completed survey was mailed back.

Surveys were sent out to a total of 2,514 households within the Detroit lower east side neighborhoods, with 258 households returning the survey, resulting in a 10.3% return rate. There were a total of 70 surveys removed, with a small number attributed to being outliers, but the majority due to insufficient travel or shopping data. Most of the removed cases associated with insufficient data were a result of respondents not filling out the precise locations where they shopped. For the final analysis, a total of 188 households were used for the fABM development and experimentation.

Overall, given the racial, socioeconomic, and neighborhood characteristics within these lower east side Detroit neighborhoods, the response rates were similar to other mail surveys in U.S. communities with a comparable sociodemographic composition [41–43]. It is well documented in the existing research that lower return rates can be expected in high-density urban neighborhoods, communities with high concentrations of racial minorities, high crime neighborhoods, and from individuals with fewer years of schooling [41–43].

Thus, given the sociodemographic composition and the perceived safety of residents, the lower return rates were expected. However, it remains important to recognize, and especially within this context, that the sample remains representative of the underlying population, which is an essential consideration with lower response rates [44]. Based on the U.S. Census Bureau’s American Community Survey, 2006–2010 5-year estimates [44], the collected sociodemographic variables–including race, marital status, household size, median household and personal income, sex, marital status, employment, and access to a private vehicle–were all relatively representative of the broader census tract population within the research neighborhoods.

It should also be recognized that the response rate was impacted dramatically by the subprime mortgage crisis experienced during the Great Recession, starting in 2007. Within three months, from when the addresses of occupied residences were obtained from the post office, to when the random samples were selected, and the surveys sent, approximately 700 of the selected homes were vacated, with no surveys returned from these households. The nature of abandonment, vacancies, and foreclosures in our study site during this period is also revealed through changing densities over the 2000 to 2010 decade. In 2000, the neighborhoods composing our study site maintained densities of 6,314 people per square mile [39, 45]. By 2010, these same neighborhoods maintained densities of 3,928 people per square mile, illustrating the level of abandonment [36, 39].

It should also be recognized that the accelerated disinvestment resulting from the Great Recession was not only evident in the real estate market. In 2007, the last major supermarket chain in Detroit (Farmer Jack) closed at a time when the city’s population was approximately 750,000 people. Thus, the surveys were mailed-out, completed, and returned during a period that the City of Detroit did not have a single major retail national/regional supermarket chain within its jurisdictional boundaries. The supermarkets mapped in Fig 3 within the neighborhood and the city are smaller independent and discount retail outlets, many of which have a more limited availability of fresh fruit and vegetable options. By 2008, therefore, the six decades of disinvestment culminated in neighborhood food environments within the City of Detroit dominated by discount and independent supermarkets, fast-food restaurants, and convenience, liquor, and dollar stores. The analysis in this research provides an imprint of the city in an extreme condition of decline, following decades of disinvestment, and a nadir in Detroit’s modern history of healthy food option offerings. In 2013, major retail chains would again return to the city, with both Meijer and Whole Foods opening stores within Detroit’s jurisdictional boundaries.

Concerning the distance values used in the analysis, there is a unique dimension to the data collection and geocoding process within this study. In the survey, we asked for the names and addresses of destinations, the store names, and the exact locations. All trip end-points were located through site surveys and mapping programs, allowing for ‘ground-truthing,’ and then the destinations were recorded and geocoded. By knowing all starting and end-points of trips, we referenced all journeys through Google maps, using the algorithm that determines the shortest time route, in-networked distance between destinations. Thus, while the respondents recorded perceived distances in the survey, by controlling the starting and end-points of trips, our analysis utilized the shortest in-network distance to minimize errors associated with self-reported perceived distances. It should be recognized that there were significant differences between self-reported perceived distances and the shortest in-network distances. In the end, the survey data, along with the shortest in-network distances, were used to devise the agent-based model.

## Data: Agent groups, preferred stores, and other analytics

Our agent-based model (fABM, f: food) is composed of household agents (HHAs) that occupy a digital replica of the neighborhood and perform weekly trips to do grocery shopping. Based on their social status, HHAs have variable access to cars. Based on previous research and the analysis of the data used for the fABM, we established that individuals who have a car would choose a car to visit a store [6, 12]. Those HHAs who do not own a car, need to resort to other forms of transportation to buy food: walking, biking, and public transit. The HHA decision-making takes into account the type of food to purchase, store preferences, and the selection of travel mode. An emergent property of the system is the total distance traveled in a particular week, as well as the spatial distribution of grocery shopping.

The fABM is empirically-driven; hence we started from survey data analysis followed by model conceptualization. Since the urban population is inherently stratified, we decided to identify types within the respondents characterized by certain sociodemographic distributions. These types are further translated into groups of different HHAs, effectively creating agent groups. While the attributes of the whole HHA population remained the same, the distributions of these attributes differed significantly between groups. For example, the number of weekly trips made by the elderly was much lower than by the younger population. When dealing with empirical data, agent grouping was necessary to account for collinearities between attribute values. Based on previous research, we used household income and householder age as the criteria for stratification [6, 30]. The final groups are presented in Table 1. For more details on stratification, see S1 Appendix.

**Table 1:** Agent groups for different combinations of age and income. Group size in parentheses. Total sample N = 188.

Income\Age	18–44	45–64	65+
< $20,000	**CoLwInYg**	**CoLwInMiAg**	**CoLwInOld**
	Core Low Income Young (33)	Core Low Income Middle Age (43)	Core Low Income Old (15)
20,000-$49,999	**LwInYg**	**LwInMiAg**	**LwInOld**
	Low Income Young (23)	Low Income Middle Age (28)	Low Income Old (11)
= or > $50,000	**MeInYg**	**MeInMiAgOld**	
	Medium Income Young (13)	Medium Income Middle Age and Old (22)	

Data were also collected on the number of people in the household that the income supports. There is not much of a distinction in these figures as we move between the different income groups. In the ‘Less than $20,000’ category, the average number of people supported with this household income is 2.20. In the next group, the ‘$20,000 - $49,999’ household income category, there are 2.37 people supported on average. Finally, in the ‘Greater than $50,000’ category, there are on average 2.56 people in these households supported by this income.

### Preferred stores

Two spatial aspects affect the distance traveled–the location of an agent and the location of a store. The store location was obtained from the survey. We were interested in both the spatial distribution of the stores for each group, and the location of a store within three distinct shopping zones–the neighborhood (the case study area), the city (within the Detroit jurisdictional boundaries), and the suburbs (Fig 3).

**Fig 4:**
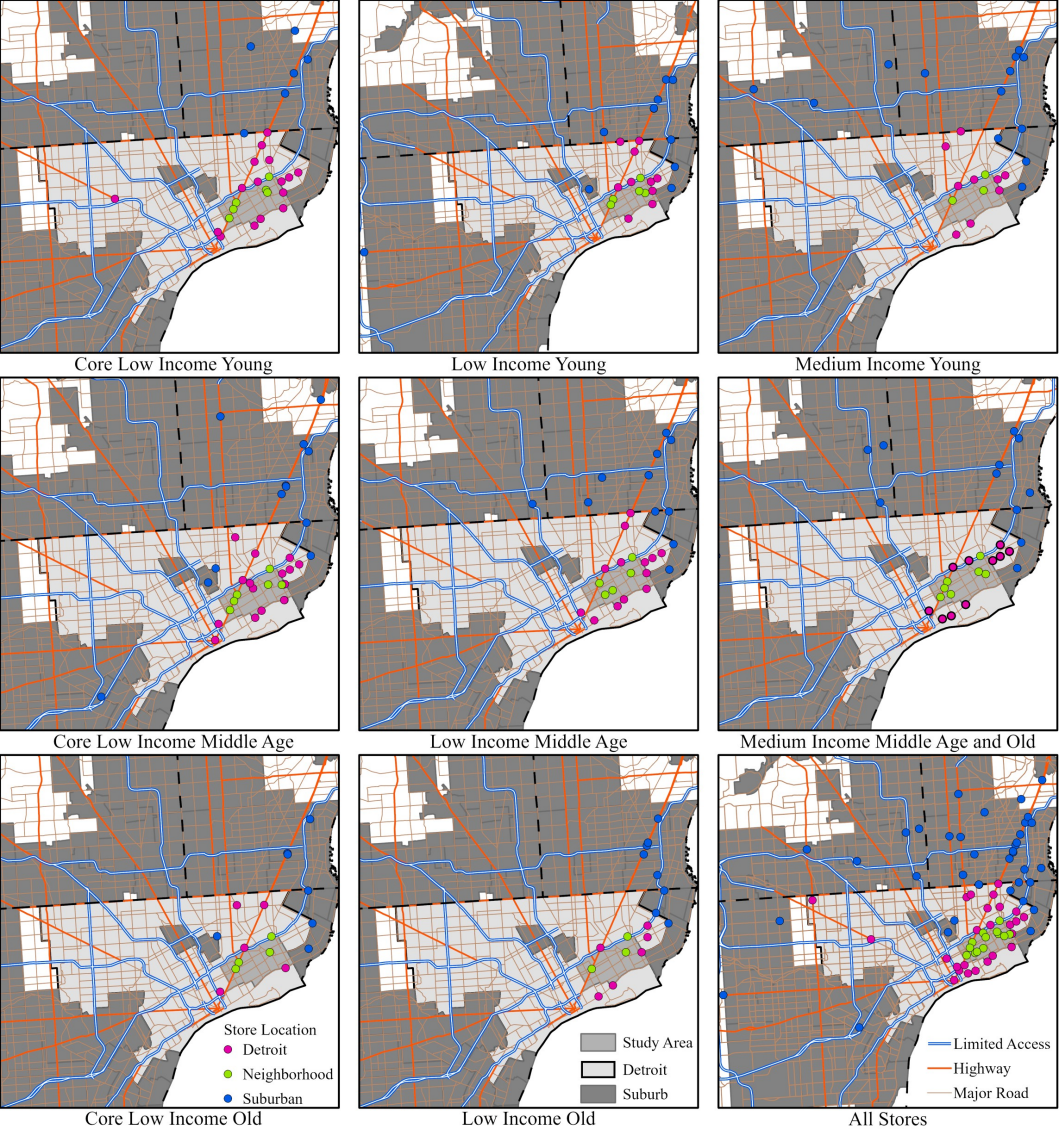
Stores identified by respondents for each agent group and their location in relation to the case study neighborhoods, the City of Detroit, and the suburbs. See Table 1 for group names. Map generated by authors, using public domain data from [39] under a CC BY license.

Fig 4 shows the spatial distribution of the preferred stores identified by respondents in each age/income subgroup. Overall, visited stores tend to be located along Gratiot Ave (Figs 3 and 4), with other clusters either far in the suburbs or in the vicinity of the case study neighborhoods. Also, “medium income” populations visit stores over a larger area–their shopping patterns are more dispersed. In contrast, the distribution of stores for the “old” population groups is more clustered. When we partition the stores into the three distinct shopping zones: the neighborhood, the city, and the suburbs, we can observe that the overall distribution of stores within zones is similar for all eight groups (Fig 4). The general trend, regardless of the age/income stratification, is that shopping in stores takes place outside of the case study neighborhoods–either in different parts of the City of Detroit or in the suburbs.

### Other data

To obtain population-level results, we upscaled the sample to the Census Bureau household population estimates obtained from ACS for census tracts, 2006–2010 [44]. We used the sample-based Monte Carlo proportional upscaling according to the guidelines in Smajgl et al. [46]. The upscaling was validated against results reported in LeDoux and Vojnovic [30]. The agent population amounted to a little over 14.5k HHAs. Agents were placed in a GIS-based environment composed of houses, stores, and a road network. HHAs were allocated to houses based on agent group distributions within the census tracts. Using the road network datasets, we calculated an origin-destination table between every house and every store in the area. This table was used to retrieve network-traversal distance (in miles) after HHAs had decided which store to visit.

## Agent-based model

As mentioned above, the fABM is composed of household agents (HHAs) that operate in an environment built from houses, roads, and stores. At setup, houses in every census tract are randomly populated by HHAs according to agent group distributions in a given tract. The model is then run in weekly intervals. Below is an overview of the grocery shopping behavior. For details about the model, see its ODD description [47, 48] in S1 Appendix.

**Fig 5:**
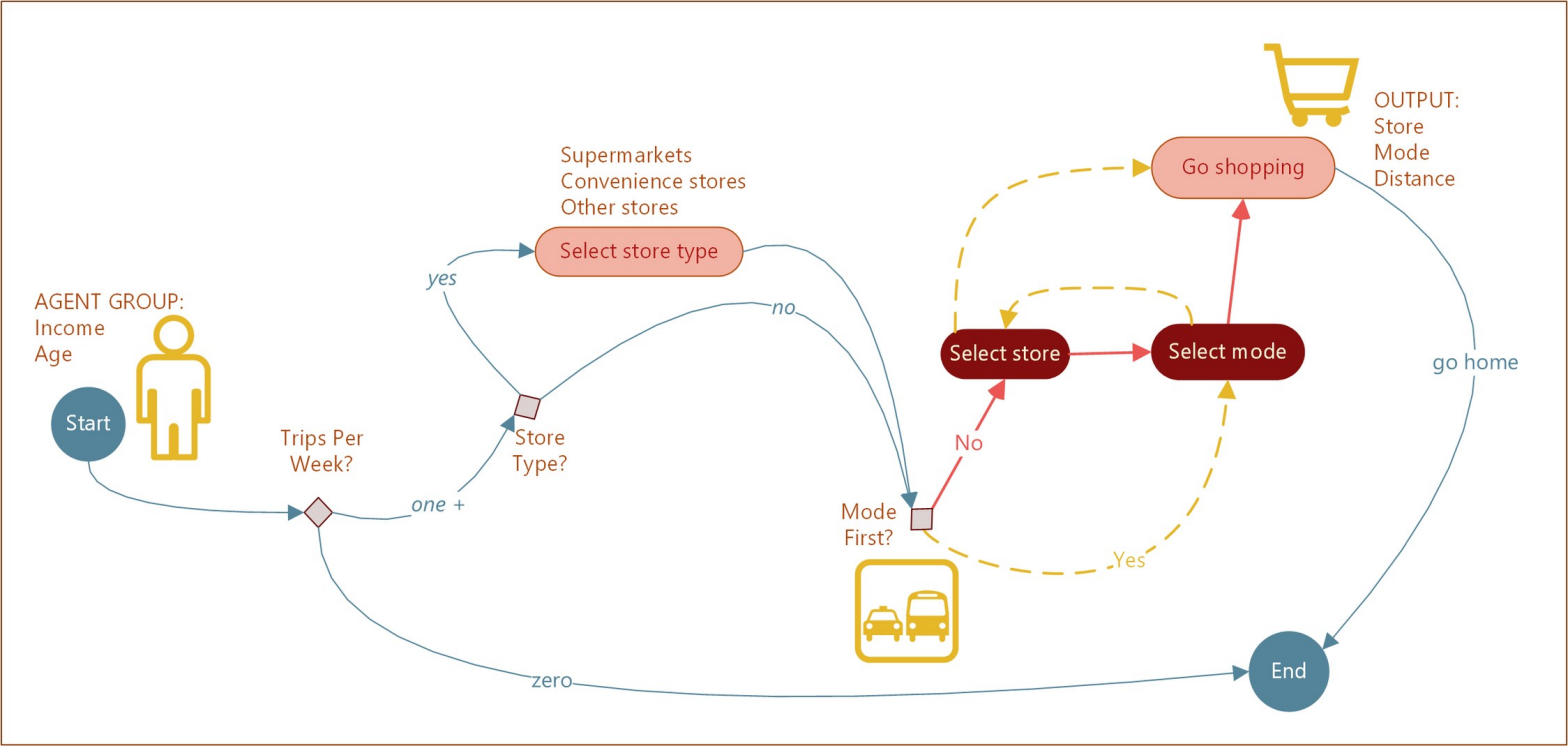
Household agent grocery shopping decision-making.

The decision process of grocery shopping is presented in Fig 5. Every week, a HHA decides on the number of store visits. For every trip, the agent then decides whether or not to consider a store type in the trip decision. In the fABM, store type is a proxy for what groceries to buy. The next step is to decide whether the trip is mainly based on the mode of transportation or the preferred store. If a mode is selected first, then the next choice is a store and vice versa. After the selection of store/mode, the agent visits the desired store and purchases food.

While the whole ABM is derived from survey data, the decision process only partially reflects empirical observations. In particular, we were uncertain which was the primary driver of the final decision–the selection of a store or the mode. Note that the socioeconomic status is essential in making the decisions regarding store selection and the mode of travel since the core poor (under $20,000 in income) are about three times more likely not to have automobile access, which, in turn, impacts their choices on how and where to travel for shopping.

The two options can be described as follows:

1. The mode first, followed by the store: *I have a car at my disposal*, *so I can go to (my favorite) Aldi supermarket to purchase veggies*.
2. The store first, followed by the mode: *I’m going to (my favorite) Aldi supermarket*. *Since I have a car*, *I’ll drive there*.

Note that, in either case, store/mode selection is based on probability distributions obtained from survey responses. Hence, if a given agent group has more access to cars, cars will be used as a primary mode of transportation. Consequently, the store/mode choice is not entirely random–it depends on the distribution of car ownership and the number of motorized trips (out of total trips) reported by the respondents. To evaluate how the order of store/mode steps affects the results, we performed a variance-based global sensitivity analysis (SA) of the fABM. Based on the results of the SA, we concluded that, for the main output of the model, i.e., median distance traveled, the order of store/mode had a secondary influence on the variability of the outcome. For all agent groups, the major contributor to the variability of the distance traveled is the decision on whether to include the type of store in store selection. Indirectly then, we can conclude that HHAs were first and foremost interested in the type of groceries to buy, followed by the store/mode choices.

Due to the lack of secondary data, we used the model-to-model (M2M) approach for the fABM validation [49, 50]. Specifically, we used conclusions derived from the statistical models of the survey data reported in LeDoux and Vojnovic [30]. This partial-validation allowed us to conclude that the fABM was suitable for application and scenario analysis.

## Results

We need to begin with the basic recognition that there are specific attributes associated with communities experiencing disinvestment, which generate unique travel costs (temporal and monetary) to the local population. The exodus of major supermarkets has produced uneven food environments, where the limited store options within these neighborhoods force all residents to travel long distances to access major retail outlets. An added consideration within these discussions is that Detroit is a typical American, automobile-oriented city, where motorized transport dominates all other travel modes and the public transit infrastructure has been long neglected. Acknowledging these particular characteristics and burdens faced by Detroit’s lower east side residents, we examine travel behavior among the different sociodemographic subgroups, with the emphasis placed in this analysis on examining the impact of age and income in shaping the nature of food-related shopping.

**Fig 6:**
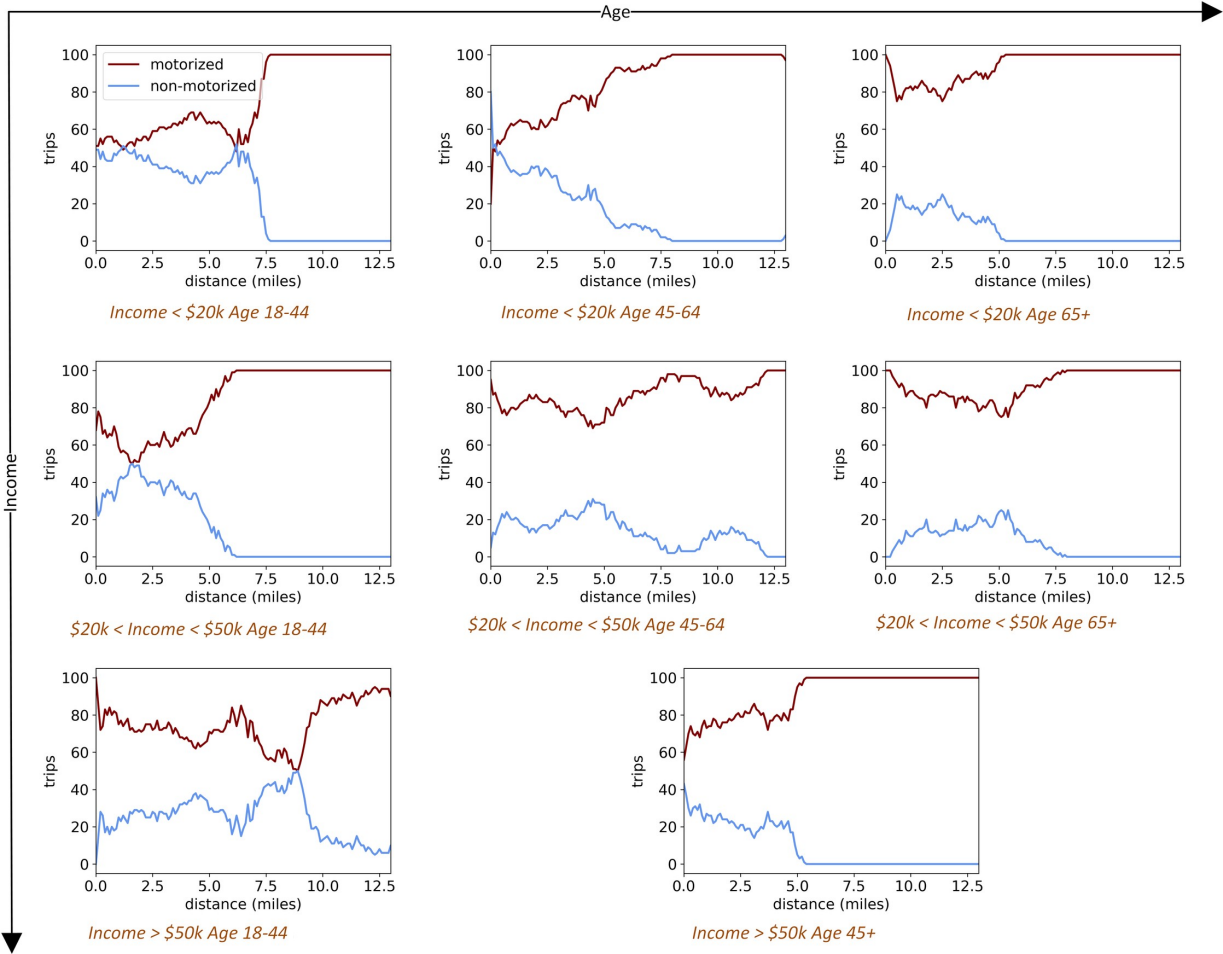
Sociodemographic subgroups and trips by mode by distance. See Table 1 for group names.

While a higher percentage of non-motorized travel in reaching shopping destinations is more likely to be observed among the young, it is much lower for the elderly (compare 1^st^ and 3^rd^ column in Fig 6), who tend to travel more by motorized means, regardless of income. In general, the younger population maintains higher frequencies of food-shopping travel, which is also more varied and dynamic–in terms of shopping locations, distances, and mode–when compared to all other sociodemographic groups. The lowest-income segment of the “young” population group (the core poor making less than $20,000 in annual household income), will average between 40 and 50% of their trips using non-motorized means. Notice that this might involve travel patterns such as walking to the store, then taking a taxi or jitney back home. Walking and cycling remain significant modes of travel for reaching stores up to 7.5 miles from their homes. In comparison, for the “low income young” (making between $20,000 to $49,999 in annual household income), their shopping trips are slightly more skewed toward motorized travel when compared to the “core low income, young” group.

Nevertheless, for the “low income young,” there is a relatively significant proportion of trips by non-motorized means for reaching stores located at a distance of up to 3 miles. Motorized travel to stores steadily grows beyond the 3-mile distance, with journeys by cars and buses becoming exclusive for stores located at 6 miles or further from home. It should be recognized that, while walking and cycling are grouped as part of non-motorized travel, it can be assumed that cycling becomes more prevalent as distances to stores increase.

**Fig 7:**
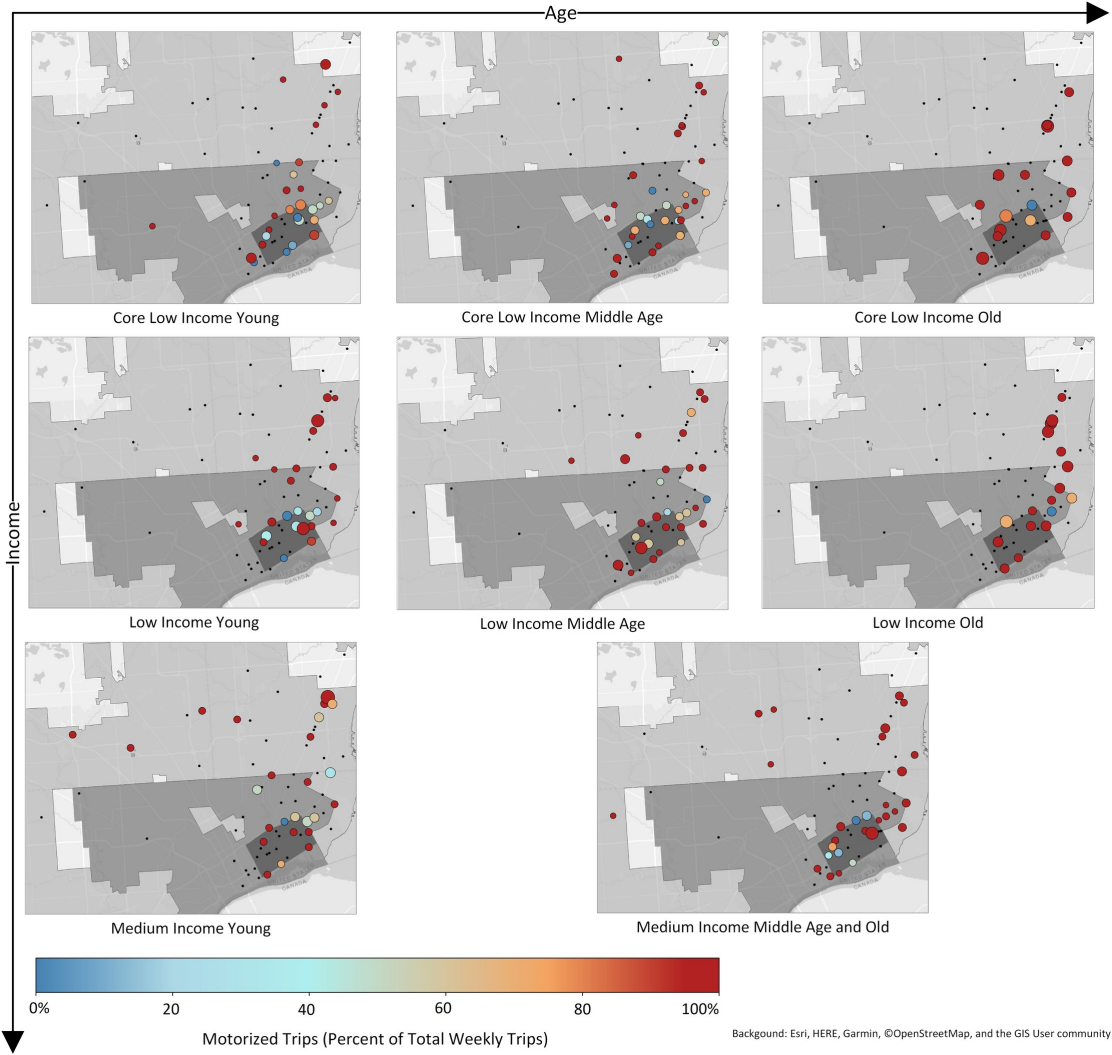
Distribution of store visits and percent of motorized trips. The larger the circle for a store, the more weekly visits made to that store. Results were normalized across all groups. See Table 1 for group names.

The most complicated travel pattern to stores is for the “medium-income young” subpopulation group (with annual household incomes of $50,000 and higher). Motorized means dominate shopping-related travel across all distances for this group. This can be expected since access to a car increases with higher incomes. Even the short trips that they make to stores close-by their homes tend to be by motorized means, generally by car. At the same time, some individuals among the “medium-income young” rely on non-motorized shopping-related travel, even to stores that are at a distance of more than 8 miles away, with a leveling off in terms of motorized travel beyond this point. Once again, some of this travel might be by cycling, but it might also be walking one way to the store and then taking a taxi back home. The “medium-income young” is a population subgroup whose food-shopping patterns across the region will become better understood in the discussion of store selection, as evident in the mapping of retail travel (see Fig 7).

Generally, the other age-income population subgroups tend to be similar to each other, with the percent of non-motorized trips to reach shopping destinations among them falling within the 0 to 20% range. There is a slight difference for the “core, low-income middle age” group, where non-motorized travel remains relatively high, between 20% to 40% for reaching stores up to a distance of 5 miles. Overall, though, as noted earlier, Detroit residents are highly reliant on the automobile across all sociodemographic groups, a typical condition of American cities. Even among low-income populations, motorized travel remains vital for trips to retail outlets, and especially when travelling to stores in the suburbs. But a clear pattern is apparent, with the young and the poor walking and cycling to stores more often than other groups. Meanwhile, the elderly generally utilize motorized travel for shopping, regardless of income.

While there are distinct mode selection and distance to store food-shopping patterns among the different population subgroups, it is essential to remember that each group has a unique set of retail outlets that are identified by their respondents as shopping locations. Individual group travel profiles cannot be understood without the spatial context, shown in Fig 7. As in Figs 3 and 4, the maps are divided into three shopping zones: i) the case study neighborhoods, ii) the City of Detroit, and iii) the Detroit suburbs. If a store is located within the neighborhood or the City of Detroit’s jurisdictional boundaries, it will be classified as close to home, and “shopping trips” will fall closer to zero miles on the distance charts above. If stores “nearby” are equally visited by car/bus and by bike/walk, and they are frequently visited (larger circle), they will weigh-in more to the MOTO/NON-MOTO ratio balance (light blue/light pink) at a particular distance (Fig 7).

The maps reflect and confirm the above distance/travel mode chart data, but they add a spatial dimension to food-shopping related travel. The maps allow us to examine and identify spatial heterogeneity in travel modes by specific locations. They reveal that there is more spatial heterogeneity in the MOTO/NON-MOTO variable among the “young”–especially in the “core low-income” and “low-income” groups (more colors for stores from the color-scale continuum)–when compared to the “old population” subgroups. Also, there is observable spatial heterogeneity in the frequency of visits to specific stores, with distinct preferences and hot spots emerging by age and income.

For example, if we just compare the shopping patterns of the “young” population subgroups, there are considerable distinctions in shopping preferences by income. Generally, all follow the major thoroughfares for reaching the frequently visited destinations. A critical transport and shopping corridor for Detroit’s lower east side neighborhood residents is Gratiot Avenue. Some of the most frequented stores along the Gratiot Avenue corridor include two Kroger supermarkets, a Meijer supermarket, and a Sam’s Club, all located in the suburbs, and all three retailers being major national/regional supermarkets.

Gratiot Avenue is an arterial that not only accommodates high capacity automobile flows but also maintains a bus line that extends through the case study neighborhoods, running into the northeast part of the City of Detroit and the suburbs. At the time of the survey, the residents would need to exit the Detroit bus at the city boundary, and wait for another bus to take them further into the suburbs in order to access the more distant supermarkets. Given the bus line, a wide range of income groups are accommodated in utilizing motorized travel to reach shopping destinations along Gratiot Avenue. The preferred shops by our neighborhood residents are located within the suburbs along this corridor, and they emerge as major shopping nodes and hotspots for the different sociodemographic groups. This is evident in the examination of the young population subgroups alone when evaluating their shopping patterns and examining how they vary with income.

If the food shopping patterns of the “young” are explored along the Gratiot Avenue corridor, considerable variations are evident depending on income. For the “core low income young,” while some of their shopping takes place within the case study neighborhoods (25% of trips), they have a significant number of shopping nodes in the city (over 50%), and a few in the suburbs (about 18%), including a single distant hotspot, the furthest out location among the three “young” income groups. In contrast, while the “low income young” maintain a few clusters in the northeast along Gratiot Avenue–with more significant nodes than the “core low income young”–their hotspot is still in the suburbs, but closer to the case study neighborhoods. However, the “low income young” frequent stores in the suburbs at twice the rate when compared to the “core low income young.”

The shopping patterns along Gratiot Avenue among the “middle income young” consist of a few nodes, and particularly in the more distant suburban locations, with their hotspot also in the suburbs, but between that of the “core low income young” and the “low income young”. Also, “middle income young” have several shopping nodes spanning across the north and the northwest suburbs. While the “core low income young” and the “low income young” have a number of shopping nodes and hotspots within the case study neighborhoods, the “middle income young” do not engage in much shopping within the vicinity of their homes. Only about 11% of their weekly shopping trips are within the lower east side neighborhoods. Overall, the “middle income young” maintain an expansive spatial extent in shopping, with a high frequency of shopping taking place in the suburbs. The “middle income young” shop over six times more every week in the suburbs compared to the “core low income young.”

At the other end of the age spectrum, if the shopping patterns of the elderly are examined, they have a much more constrained set of regularly visited stores forming a group of equally visited shopping nodes. Still, they travel for food shopping far less frequently when compared to the young. But even among this sociodemographic subgroup, which travels the least overall for food shopping, more shopping outside the neighborhood is evident as incomes increase. In general, higher incomes among sociodemographic groups allow more freedom to travel, and this is apparent in their wider expanse of shopping trips across the Detroit suburbs, see Fig 7.

The distance/travel mode chart data among the “medium income young” is highly varying and dynamic, Fig 6. This group, in general, relies extensively on their automobiles, but also maintains a consistent pattern of walking and cycling–shaped by a variety of distinct behaviors in trip generation among its members. Overall, the “medium income young” is the group, which, by far, makes the most weekly shopping trips. This group makes short in-neighborhood motorized trips for shopping, i.e. a pattern of driving to close-by stores. This sociodemographic group also maintains a consistent proportion of non-motorized visits to stores at the border of the neighborhoods, just outside of the northeast case study boundaries, but still within the City of Detroit.

There is a population subgroup among the “medium income young” who are more disposed to walking and cycling to nearby stores. This is not surprising since, even among this highest income sociodemographic group within Detroit, there are households without access to an automobile, about 10%. Within this income bracket, there may be a segment of the young and the wealthier who on cultural and socio-political grounds have decided to simply live without a car. This could be a segment of young higher-income earners who are committed to non-motorized travel, being fully devoted to biking and walking to their daily destinations.

In terms of the broader travel patterns for food shopping among the “medium income young,” the emphasis continues to be placed on frequenting stores running along the northeast corridors from the case study neighborhoods. These include major supermarkets along Gratiot Avenue, but also, the second- and third-tier shopping strips, like stores off the Edsel Ford Highway along Harper Avenue, Kelly Road, E. Warren Avenue, Mack Avenue, and East Jefferson Avenue. Moving further northeast away from their neighborhoods, yet still within the city, the “medium income young” maintain a substantial number of motorized trips to shops at the City of Detroit border. More significantly, however, in a dynamic pattern covering a greater spatial area than any other income group among the young, their personal shopping preferences, relative wealth, and access to private vehicles allows them to emphasize shopping in the suburbs (over 50% of the food shopping trips).

As one might expect, for the “medium income young,” shopping in the suburbs is dominated by motorized travel, although there are three stores outside the City of Detroit boundaries that are regularly accessed by non-motorized means. Two of these are along Gratiot Avenue. Shopping corridors, such as Gratiot Avenue, emerge as important shopping destinations for non-motorized travelers. Those corridors offer pedestrians and cyclists comparatively shorter distances between the different store destinations, as they move along this arterial. The increasing number of destination locations is more of a draw for non-motorized shoppers simply because of the more regular and relatively shorter-distanced store options along Gratiot. The case of Gratiot Avenue also speaks to the importance of arterials that offer and accommodate a balance between different modes of travel. This corridor accommodates high capacity automobile flows, a bus line, and also comparatively more destinations for pedestrians and cyclists, with ongoing and regular store options extending from the City of Detroit and into the suburbs.

For the case study neighborhoods as a whole, while Gratiot Avenue (see Fig 3) emerges as an important retail strip, there are several other arterials, all extending northeast from the case study neighborhoods and into the suburbs, that serve as major shopping destinations for locals. A secondary major corridor emerges along Harper Avenue, which runs parallel along the Edsel Ford Expressway, starting within the case study neighborhoods, moving northeast across the City of Detroit and into the suburbs. Four other, lower-order arterials serve as shopping nodes for the lower east side neighborhood residents, including Kelly Road, E Warren Avenue, Mack Avenue, and East Jefferson Avenue. All of these major shopping corridors run through the lower east side Detroit case study neighborhoods and extend east and northeast across the city and into the suburbs, with many regularly visited shopping locations across all three distinct regional shopping zones.

## Discussion

In the post-Great Recession era, considerable international media attention was focused on disinvestment in the City of Detroit, the breakdown of local public transportation, and the difficulty that the residents faced in accessing essential daily destinations, including healthy food options. One story that attracted major international attention throughout 2015 and 2016 focused on 56-year old Detroit resident James Robertson, who walked a 21-mile round trip daily between work and home, in the sun, rain, and/or snow [51]. This case effectively speaks to the scale of urban disinvestment and commercial suburbanization across the Detroit region, along with the resulting consequences on local mobility. His total trip was 23 miles, with Robertson taking a two-mile bus ride partway over his journey, across a corridor where he had access to public transit. It should be recognized that these types of mobility burdens among Detroit residents are not unique. As evident in this research, due to the scale of disinvestment within the city, the concentrated poverty, the lack of access to private vehicles, and the inadequate local public transit, low-income Detroit residents spend considerable time travelling to reach basic daily destinations, in many cases by non-motorized means.

This analysis examines the complex nature of food-shopping related travel in landscapes of disinvestment and uneven food environments, where due to the low incomes, large segments of the population do not have access to a car. Yet, they live in communities where there are few options within the neighborhood, or in reasonable proximity, to walk and purchase groceries. Due to the nature of disinvestment and the loss of major neighborhood supermarkets–a result, in part, of the decades of suburbanization–this research explores a unique urban environment, where residents have little choice but to travel long distances to access a full array of nutritious food options. The typical neighborhood resident makes about five shopping trips per week, traveling an average of over five miles to reach each store. This is evident in the research results, with the vast majority of grocery trips for local Detroit residents taking place not only outside of their neighborhoods but beyond the boundaries of the city. In fact, much of the food-shopping by Detroit’s lower east side residents (more than one-third), takes place in the suburbs, as the local population travels considerable distances to reach major national/regional supermarket chains, including Kroger, Meijer and Sam’s Club. Also, the analysis of these 23 census tracts takes place in a section of the city that comprises just under 10 square miles, yet only 23% of the shopping trips by residents within these neighborhoods takes place within this relatively large case study area in Detroit’s lower east side.

**Fig 8:**
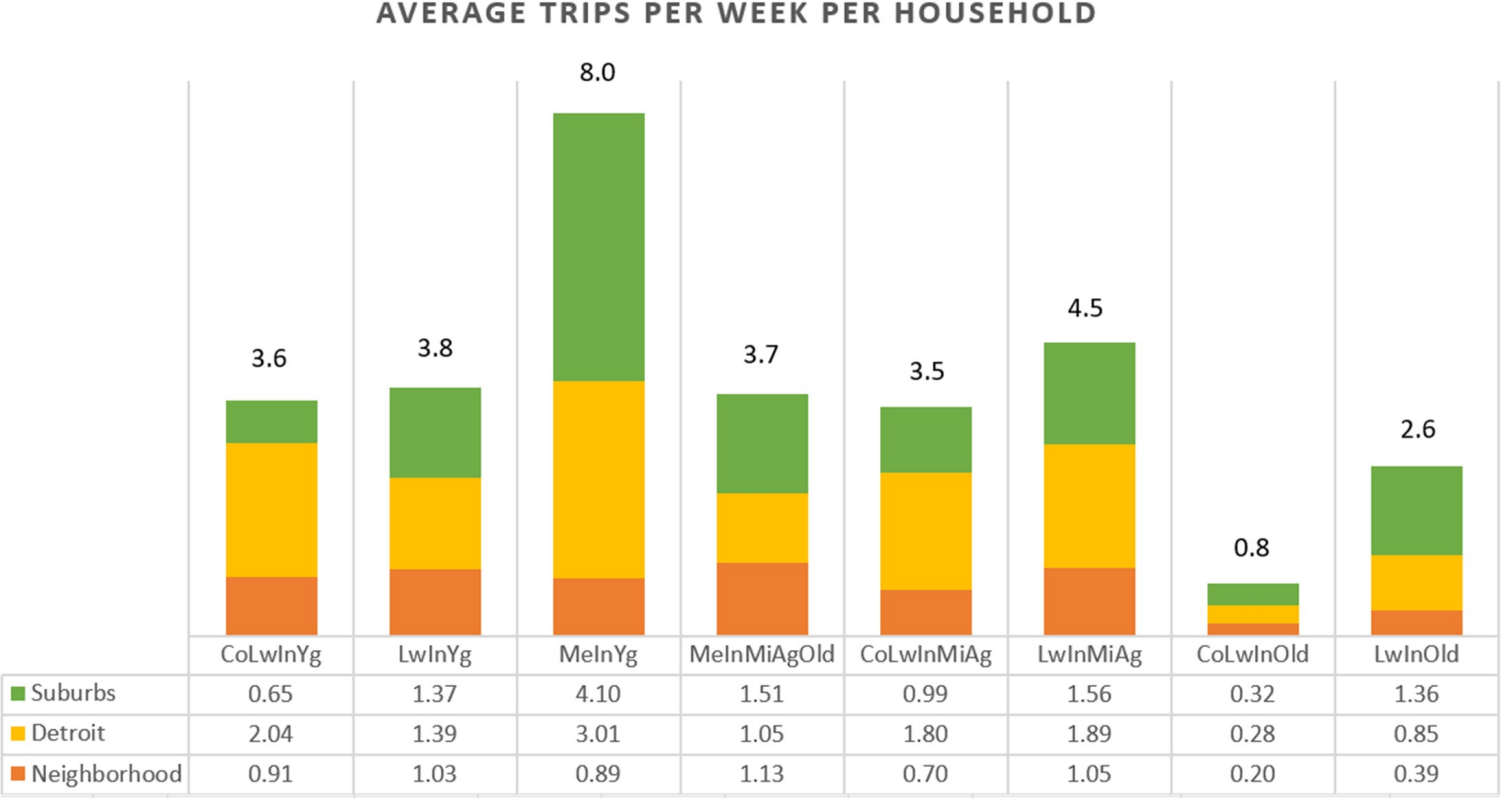
Average shopping trips per week among different sociodemographic groups and the three distinct shopping zones. For group names, see Table 1.

While these are low-income neighborhoods experiencing severe disinvestment, and while the residents travel extensive distances to buy food–due, in part, to the absence of major national/regional supermarket chains within their neighborhoods, or in close proximity–the different sociodemographic groups still maintain unique patterns of grocery-shopping behavior. These distinctions can be seen in Fig 8, showing frequencies of shopping visits by the three different shopping zones (the case study neighborhoods, the City of Detroit, and the suburbs), by the distinct sociodemographic groups. In shaping food-related shopping behavior, the emphasis continues to be placed on age (with particularly the “young” traveling more than the “old”) and income (with higher incomes resulting in greater automobile access, thereby allowing more frequent travel to farther suburban locations).

Also, the research results show that there are different thresholds of tolerance for non-motorized travel to stores that are shaped by sociodemographic patterns. In neighborhoods of disinvestment and among the poor, these tolerance thresholds are an outcome of necessity. As shown in this analysis, there are implications associated with variations in income and age in shaping food-shopping trip patterns. Younger and poorer population subgroups will have a higher propensity to walk or cycle for shopping, and for the lower-income populations, this is generally a result of necessity, since they will likely not have access to a car. In comparison, older population subgroups, regardless of income, will tend to select motorized travel to reach their stores of choice. Notice that these distinctions are drawn when controlling for the built environment, with a unique built environment-controlling data collection and analysis strategy. Specifically, the data are gathered, and the analysis takes place at the neighborhood scale, in order to control for the built environment.

From a planning perspective, the success of shopping corridors, like Gratiot Avenue, that meets the needs–and offers a balance in terms of transportation options–of different modes of travel, is also reinforced by this research [7]. Facilitating different travel modes, a variable that will continue to attract stores and other amenities along these arterials, ensures the development of effective shopping corridors, whether for pedestrians, cyclists, public transit riders, or drivers. Notice that this remains the case even along thoroughfares in neighborhoods experiencing severe disinvestment. By accommodating high-capacity automobile flows, a bus line, along with extensive and regularly interspersed shopping destinations (particularly important for pedestrians and cyclists), ensures that Gratiot Avenue emerges as an active corridor for both motorized and non-motorized travel. Also, with Gratiot Avenue extending from the City of Detroit and into the suburbs, the arterial offers many different store types along a lengthy artery. It has become a significant shopping corridor with many destination hotspots for different sociodemographic groups.

It should be recognized that very little of the shopping by the Detroit lower east side residents is in more proximate Grosse Pointe to the direct east, despite the location of Kroger and Trader Joe’s at the time, both major national/regionals supermarket chains. This is in large part due to the racial hostility and the street network barriers introduced, which not only reduce interest among the Detroit residents to access this jurisdiction, but hinder their capacity to enter this eastern municipality. The Detroit residents are actively selecting to shop in Harper Woods, Roseville, and Eastpointe in the northeast, while avoiding Grosse Pointe.

The distance/travel mode charts and the distinct store food-shopping spatial patterns among the different population groups provide insight into the dynamics of travel for food shopping. They also reveal the costs to residents (temporal and monetary), living in communities experiencing disinvestment, associated with accessing stores for groceries. As a whole, Detroit’s lower east side neighborhoods offer few options within the neighborhood, or its immediate proximity, to purchase a full array of healthy foods. In addition, while there is some variability in sociodemographic composition, these are generally lower-income neighborhoods, with over one-third of the households not having access to an automobile. Nevertheless, the research results do show pronounced variability to food-shopping related travel–from average distances traveled, to the mode of travel, to the store of choice–particularly as shaped by age and income.

It should be acknowledged that these research results challenge foundational tenets in urban planning and design, regarding the specific characteristics necessary in the built environment to facilitate accessibility. It is generally argued that reducing the average distances between one’s home and necessary daily destinations requires three critical variables in the built environment. These include high residential densities, a mix of land uses (integrating residential, commercial, public, and other uses), and high-connectivity streetscapes (rich throughway networks linking different parts of a neighborhood and different neighborhoods together). However, in neighborhoods experiencing disinvestment, where urban amenities have left the neighborhood–and in some cases, as in Detroit, the city altogether–resident cannot walk to local amenities because they no longer exist in close proximity, even though the neighborhoods themselves might be characterized by relatively high densities, mixed land uses, and high-connectivity street networks. This speaks to the importance of sociodemographic conditions (class, ethnicity and race), and their relevance to trip generation, being ultimately more important than the built environment in shaping accessibility and travel behavior. An average distance to a store of over five miles for locals is not a reasonable definition of accessible.

Since these data were collected, two supermarkets have opened in the City of Detroit that could have the potential of influencing neighborhood shopping behavior. One is a Meijer store, which opened in Detroit in 2013, on Woodward Avenue and W. Eight Mile Road. While within the city limits, the store is at a minimum in-network distance of 10 miles from the closest boundary of the case study neighborhoods, and closer to an average in-network distance of some 13 miles for the typical residents living in our study area. There are far closer supermarket options along Gratiot Avenue for the neighborhood residents–including another Meijer supermarket–which not only have the benefit of a more direct, single-route public transit line, but also the grouping of other proximate supermarkets all along this corridor. The medium income residents might venture and shop at the new Meijer location, because of their more dynamic travel and food shopping behavior. But the medium income earners–like the lower income residents within the case study neighborhoods–do have access to the same store in closer proximity, and the benefit of other clustered retail locations nearby.

A Whole Foods Supermarket also opened in the City of Detroit in 2013. It is located in the area of Midtown Detroit, at Woodward Avenue and Mack Avenue, and this would be a relatively close shopping option for our neighborhood residents. The Whole Foods Supermarket, in Midtown, is at a minimum in-network distance of 3.25 miles from the closest boundary of the study area. For the typical residents living in the case study neighborhoods, they would travel an average in-network distance of 5.6 miles to reach this destination. Whole Foods, however, is recognized as selling very expensive food options [52]. The opening of Whole Foods in Midtown Detroit is largely marketed to the various Medical centers, Wayne State University, and the high-end condos that are gentrifying this area of the city.

Given the prices of food and the socio-demographic make-up of residents, at best, Whole Foods might appeal to the highest income bracket within the case study neighborhoods, the medium income groups. Given their more varied and dynamic travel and food shopping behavior, some medium income residents might find Whole Foods attractive, but again, the high prices within this supermarket will likely discourage many lower east side residents from regularly shopping at this location. Particularly, given the low number of medium income earners–recall the case study neighborhoods maintain median household incomes of $20,882 annually–Whole Foods is unlikely to dramatically change the travel to store patterns for food shopping among our lower east side residents. Nevertheless, some medium income households might find this shopping option attractive.

### Limitations and future directions

Unlike other food access ABMs, which are designed to evaluate a spectrum of policies, our model was developed to address a particular research question in an applied context. In future fABM versions, we will continue to introduce dimensions that reflect the essential aspects of a healthy and sustainable urban food system, especially for disadvantaged communities. In the next iteration of the fABM, we will include a health component to further study the relationship between store selection and the distance traveled to purchase both low quality and nutritious food. This will require expanding the model with other actors of the system–especially grocery shop owners who make decisions about the assortment of food products offered in the store. We also acknowledge that the model targets a very specific population. In this respect, it is not transferrable–in its current form–to higher-income, heterogeneous, robust and more dynamic communities that maintain a full array of grocery store options and other urban amenities. We would also like to explore how gender and education shape grocery shopping behavior. Finally, since the model was only validated using another model as a baseline, its applicability in the current form is limited. We need other modes of validation to enhance the fABM accuracy. Of particular interest here are participatory approaches to ABM [29, 53, 54] where the impoverished communities in our study area take active part in model conceptualization and reflect on its current implementation, suggesting future improvements or pointing out existing deficiencies.

## Conclusions

In this research on the dynamics of food-shopping behavior, the study provides a unique contribution to complex urban systems modeling, and specifically agent-based models (ABMs), by utilizing real-world data in simulating individual travel behavior in accessing food in low-income Detroit neighborhoods experiencing disinvestment. The significance of the model lies in modeling travel behavior based on store preferences identified in a survey. The agents operate on a road network, which allows for a realistic measurement of home-store distance, and identification of the mode of transportation based on sociodemographic characteristics of the respondents. The case study neighborhoods are in Detroit’s lower east side, with the data collected during a period when the city did not have a single major national or regional supermarket chain within its boundaries. The neighborhoods are predominantly occupied by African American communities (about 94%), where only 6.4% of the residents hold a university degree, and fewer than 18% of the residents are married. Residents within these neighborhoods maintain a median household income of $20,822, and about a third do not own a car. On average, they travel over five miles to get to a store for their grocery shopping, illustrating the travel burdens that locals face in reaching basic daily necessities, such as healthy foods.

In examining neighborhoods experiencing severe disinvestment and decline, the research focuses on food access that is constrained by both physical space and class status. Yet, despite associated limitations to mobility, patterns in food shopping do emerge by sociodemographic composition, particularly shaped by age and income. The choice of stores, the number of weekly trips, the use of different travel modes, and the distances of the weekly journeys devoted to grocery shopping all take-on distinct patterns by sociodemographic composition, illustrating the complex nature in grocery-shopping travel behavior in uneven food environments.

This research reveals that there are different thresholds of tolerance for non-motorized travel to stores that are shaped by sociodemographic characteristics. Although, it should be recognized that in neighborhoods experiencing decline, where large segments of the population are living in poverty, non-motorized travel is, in large part, an outcome of necessity. With the research focused on neighborhoods experiencing extreme disinvestment, the study illustrates how income and age, in particular, influence the probability of whether one selects motorized or non-motorized travel for grocery shopping. The research results show that the younger and the poorer population subgroups will have a higher propensity to utilize non-motorized travel for shopping. The most dynamic travel patterns in grocery shopping are carried out by the “medium income young” population subgroup, who travel the greatest distances, across the most expansive area throughout the city and the suburbs, and shop most frequently for food throughout the week. More than 50% of the shopping trips among the “medium income young” are in the suburbs.

However, there is also a population segment among the “medium income young” who do not own a personal vehicle and who prefer to shop by walking and cycling. Given the income levels, we are likely capturing distinctions in cultural values among this population, with a subgroup of young medium income earners who are pursuing alternative lifestyles. These could be wealthier young professionals–such as teachers, university professors, and/or municipal officials–who select to live in the City of Detroit and who prefer to travel by non-motorized means, even over relatively extensive distances.

In contrast, older population subgroups traveled the least for food shopping, and when they did, they generally relied on travel by motorized means to reach their retail destinations. But even among the elderly, as incomes increased, they would engage in food shopping at greater distances from their neighborhoods. Across all sociodemographic subgroups, higher incomes allowed more freedom to travel across a greater expanse of the city and the suburbs to reach shopping destinations. Also, with the higher incomes and with a greater probability of owning a private automobile, the residents increasingly selected to shop at major regional/national supermarket chains in the suburbs, with some of the main stores of choice among the lower east side Detroit residents including Meijer, Kroger and Sam’s Club.

It should be recognized that the COVID-19 pandemic further accentuated the severe racial and class disparities across the region, with the City of Detroit’s African American community confronting disproportionate burdens. Despite Detroit having only 6.7% of the population in the state, the city had about a quarter of Michigan COVID-19 cases [55]. In addition, hospitalizations and deaths due to COVID-19 were significantly higher among the African American population when compared to the white population. Combined with the health impacts of the outbreak, there were also more severe repercussions on local mobility among the low-income Detroit residents. The reliance on buses among Detroit’s low-income population, as evident among residents in the case study neighborhoods who travelled by public transit to distant suburban stores, was dramatically impacted by the COVID-19 outbreak. This was a direct result of the health risks of using public transit, with transit ridership falling significantly below normal demand levels in the city, down 37%, during the pandemic [56]. With low-incomes, limited private automobile access, reliance on public transit for commuting over long distances, and the rapid spread of COVID-19 infections across Detroit, there was a severe overall impact on accessing stores, and this was reflected in local commercial spending. Following the COVID-19 outbreak, the City of Detroit confronted the fourth sharpest decline among major U.S. cities in local shopping, behind San Francisco, Chicago and New York, reflective, in part, on the overall mobility, sociodemographic composition, and health vulnerabilities of the population [57].

What is also captured by the modeling exercise, from a land use and transportation perspective, is the spatial dimension of food shopping and the importance of retail corridors that are effective in supporting a mix of travel mode options. The important shopping arterials that emerge from this analysis are corridors that effectively accommodate high capacity automobile flows, offer public transit, and are comparatively more attractive retail destination strips for pedestrians and cyclists due to the regular interval of stores at reasonable distances along the thoroughfares. Five major shopping corridors emerge. All are highly frequented shopping arterials that are effective in supporting a mix of travel mode options, from the bus to the car, to walking and cycling. This is an outcome expected from urban planning and design theory, which emphasizes a balance between different modes of travel in supporting dynamic corridors. Still, this relationship is reinforced in our ABM, which is unique in that it utilizes real-world individual-level travel behavior data to reveal and explain heterogeneous and dynamic destination corridors emerging in complex urban food systems.

However, the research results also challenge basic tenets in urban planning and design, which have advanced specific characteristics in the built environment necessary to promote accessibility. It has been postulated that higher densities, a mix of land uses, and high-connectivity street networks reduce average trip distances between destinations. Whether the journeys are between the home and various daily destinations (work, shop, personal services, or leisure) or between out-of-home destinations (for example, from work to shop), it has been argued that if densities are high, a finely-grained land use mix is maintained, and the street system is connected, distances will be reduced. However, these principles were established from studying relatively higher-income, heterogeneous, robust, and dynamic urban neighborhoods, such as in New York City, Boston, San Francisco, Toronto, or London.

In neighborhoods experiencing disinvestment, it becomes evident that all three of these characteristics might exist in the built environment, yet, considerable distances will exist between destinations. Because certain urban amenities have left the neighborhoods or the city altogether–such as major national/regional supermarket chains or healthy restaurant options, with both being the case in Detroit—accessibility to local necessary amenities might remain poor and trip distances to daily destinations high. These hidden community level travel behavior patterns that are unique to specific sociodemographic groups were revealed by using an ABM, which applied household-level empirical data to an artificial agent population placed in a replica of geographic space, who then actively participated in their daily shopping.

## Supporting information

S1 Appendix (DOCX)
