# Supplementary material for: The dynamics of food shopping behavior: Exploring travel patterns in low-income Detroit neighborhoods experiencing extreme disinvestment using agent-based modeling
Source: PLoS One. 2020 Dec 21;15(12):e0243501. doi: 10.1371/journal.pone.0243501 (PMC7751856; doi:10.1371/journal.pone.0243501)
Supplement: S1 Appendix — (DOCX) [file pone.0243501.s001.docx]

# S1 Appendix

**Overview, Design concepts, and Details (ODD) of fABM**

***1. Purpose***

The fABM ('f' stands for food, i.e., an ABM of food access) has been developed to explore how different sociodemographic groups utilize different modes of transportation for grocery shopping. The specific research question is as follows:

*For households grouped by age and income, how does the mode of transportation change with distance for grocery shopping in the selected study area?*

The research aims to assess how different thresholds of tolerance for non-motorized travel to stores for food shopping are shaped by sociodemographic composition. With the research focused on neighborhoods experiencing extreme disinvestment, the study examines how income and age can influence whether one selects motorized or non-motorized travel for grocery shopping.

***2. Entities, state variables, and scales***

Entities

*Agents*

1. Household Agents (HHAs) represent families in the study area. HHAs are the decision-makers in the model. Attributes:
   1. Agent Group identifier (integer)
   2. Trips per week (integer)
   3. Frequency of motorized trips (float)
   4. House (pointer to the house object)
   5. Agent Group to which HHA belongs (class)
   6. Trends – a placeholder for data collection for every trip: store visited (store ID), mode of transportation used to get to the store (1: motorized, 0: non-motorized), simulation run number, time step within the simulation
   7. Decision rule 'by mode': HHA first selects a mode to go shopping (e.g., a car), followed by the selection of the store to visit
   8. Decision rule 'by store': HHA first selects a store to visit, followed by the selection of the mode.

*Spatial Units*

1. Stores are point objects that represent store locations in the study area. Attributes:
   1. Store identifier (integer) - GIS point dataset
   2. Store name (text)
   3. Store location (geographic coordinates)
   4. Store category (text): supermarket, convenience, other
   5. Probability of selection of the store (float number within a range (0.0, 1.0])
   6. Cumulative number of trips (visits) to the store during the simulation (integer)
   7. Cumulative number of motorized trips to the store during the simulation (integer)
2. Houses are point objects that represent house locations in the study area. Attributes:
   1. House identifier (unique label)
   2. House location (geographic coordinates) - GIS point dataset
   3. House type (single-family, multi-family)
   4. Number of housing units (integer)
   5. Census tract within which the house is located (unique label)

*Environment*

1. Roads: GIS data layer (GIS network dataset). Attributes:
   1. Nodes with geographic coordinates
   2. Links between notes
2. Census Tracts (GIS polygon dataset). Attributes:
   1. Number of HHs classified by age and income (integer) based on census counts

*Collectives*

1. Agent Groups: HHAs are grouped into eight types, each with its shopping behavior. We refer to these types as Agent Groups. Attributes:
   1. Name (a label that includes both age and income categories, e.g., LwInYg is a group of low income, young population)
   2. Tracts (percentage of each census tract populated by a given Agent Group)
   3. Group identifier (text)
   4. Population (census counts)
   5. Agents – HHAs
   6. Stores – list of store objects derived from survey data

*Spatial Resolution:* Common resolution for all GIS datasets is 1:24,000 (the resolution of 'roads,' which are derived from TIGER/Line Files).

*Temporal Resolution:* A time step represents a week, and the model is executed for five weeks (~ one month).

*Location and Extent:* The model was developed for lower eastside neighborhoods in Detroit, Michigan, the U.S. It covers 23 census tracts and an area of 9.695 square miles. The stores are located within the neighborhood but also outside of it – both in the city and in the suburbs. The total area – including the stores – is 636.8 square miles.

***3. Process overview and scheduling***

Each time step (discrete time corresponding to a week) starts from HHA's decision on how many trips to make to buy groceries. The next (optional) step is to decide on the type of store to visit. This step serves as a proxy for the type of food to buy. After this optional step, HHA decides how to get to a store. This includes the choice of a specific store from a pool of stores identified from a survey. The choice is based on the frequency with which the respondent group nominated a store as their preferred one (a given store has a survey-based probability of selection). After the agent makes the trip, the model registers outputs: the store visited, the mode, and the distance traveled. This procedure is repeated for every Agent Group. The outputs are saved as *.csv files – a separate file per Agent Group.

Decision-making pseudocode:

For every week:

For every Agent Group:

For every agent:

How many trips to make?

Select the store category?

If mode initializes the trip:

Select the mode of transportation, then select a specific store

If the store initializes the trip:

Select a specific store and then select a mode of transportation

Go shopping

Update HHA's 'Trends': the store visited, the mode of transportation

The decision on store type and the order of the store/mode steps are not directly derived from the survey. First, the respondents were not asked about the store type. Instead, they simply reported their favorite stores, the frequency of visits, and the mode of travel. Since the survey was not designed with ABM in mind, it did not include questions that explicitly asked about the steps in the store selection decision. This uncertainty of decision-making was evaluated using sensitivity analysis.

***4. Design concepts***

*Basic principles.*

The fABM is almost entirely data-driven – it was informed by household mail survey data collected in the study area (LeDoux & Vojnovic, 2013). The data was then extrapolated to all households in the area using American Community Survey by U.S. Census Bureau (ACS five year estimates). Agent and store attributes have empirically derived probability distributions. For every step of the decision process, the options are selected based on these probabilities. The only completely random components are: [1] the selection of the decision rule: 'mode followed by store' or 'store followed by mode,' and [2] the allocation of an agent to a household. This uncertainty was tested by performing a variance-based global sensitivity analysis.

*Emergence*.

The distances between stores and houses are the property that emerges from the model. Distance is not considered in the decision-making process. It is retrieved at the end of the model run using an origin-destination matrix where each house and each store have a distance value in miles. The matrix was calculated using road network traversal. Other results like the number of trips per Agent Group, percent of motorized trips, and frequency of visits per store are more tightly imposed by the model because they are initialized from the empirical distributions. Given a large number of agents in the fABM, these variables are also recorded for validation.

*Adaptation*.

There are no direct adaptive mechanisms in the fABM.

*Objectives*.

Since objectives are tightly related to adaptive traits, this design concept does not apply to the fABM.

*Learning.*

There are no learning mechanisms in the model.

*Prediction*.

HHAs do not use predictive procedures to make decisions or estimate the consequences of these decisions. They simply respond to a 'stimulus' which is a value derived from a probability for a given variable. For example, certain stores are more commonly visited than others. The frequencies of visits among stores are known a priori from the surveys. Therefore, the choice of the store is strictly stochastic. This procedure repeats for every time step, and there are no mechanisms that would affect store choice based on previous time steps.

*Sensing*.

There is no direct sensing of any nature in the fABM. However, evidence has shown that the residents in the area often commute to stores. They organize one car and go shopping in small groups. While this mechanism is not coded in the model, it is implicitly present in the distributions of trips obtained from the empirical data. For instance, if two HHAs regularly go shopping together, their number of weekly trips will be similar. This can be classified as indirect sensing via a social network.

*Interaction*.

Each HHA makes the trips independently from other HHAs. Therefore, there is no direct interaction among agents. But interactions may be concealed in the probability distributions. Refer to the 'sensing' example above.

*Stochasticity*.

The fABM is highly stochastic. We did not introduce any constants (parameters) into the model. The following variables are defined using probability distributions: trips per week (separate distribution for each Agent Group), the selection of a house by HHA (random within census tracts), and store selection. Stochasticity is critical in the fABM as it reflects the real-world variability in individual household behavior, obtained from the surveys.

*Collectives*.

HHAs are put into aggregations called Agent Groups. These groups were defined using two variables: age and income (Table A1). The groups were created to account for correlations between sociodemographics, e.g., low-income families are less likely to own a car than medium-income households. Without grouping, model inputs would be sampled independently, leading to unlikely scenarios like core low-income agents always driving to buy groceries regardless of distance to the store. Each Agent Group has different probability distributions derived from the data reported by respondents belonging to the distinct age-income groups. For example, a population of households with a yearly income of less than $20,000 and the age range from 18 to 44 is classified as core low income young (CoLwInYg). Since both income and age data were divided into three classes, we ended up with nine income-age combinations. Due to the small sample size, we combined two classes into one: ‘medium income middle age’ and ‘medium income old’. After identifying distinct agent groups, we proceeded to generate distributions for each group from data obtained via the survey. For the decision algorithm, we identified three attributes: weekly trips, preferred stores (and their category), and mode of transportation, which was generalized into motorized (car, bus), and non-motorized (bike, walk). The summary of the variables is provided in Table A2. All trip distributions were positively skewed – with a significant frequency of fewer trips made weekly (~2-4) and a small frequency of many trips made weekly (> 4).

Table A1 Agent Groups in fABM. The values in the parentheses are sample size and census estimates (number of agents), respectively.

|  |  | AGE | | |
| --- | --- | --- | --- | --- |
|  |  | 18-44 | 45-64 | 65+ |
| INCOME | < $20,000 | (33, 2695)  **CoLwInYg**  *Core Low Income Young* | (43, 2455)  **CoLwInMiAg**  *Core Low Income Middle Age* | (15, 1898)  **CoLwInOld**  *Core Low Income Old* |
|  | $20,000-$49,999 | (23, 1287)  **LwInYg**  *Low Income*  *Young* | (28, 1813)  **LwInMiAg**  *Low Income*  *Middle Age* | (11, 1555)  **LwInOld**  *Low Income Old* |
|  | > $50,000 | (13, 837)  **MeInYg**  *Medium Income Young* | (22, 2148)  **MeInMiAgOld**  *Medium Income Middle Age and Old* | |

Table A2 lists the average number of stores per respondent in every group. A higher number indicates that an average representative respondent in a particular group identified more stores visited regularly. In other words, higher numbers indicate a greater number of different stores visited. Specifically, ‘core low income young’ and ‘core low income middle age’ groups reported a larger pool of preferred stores relative to the sample size (1.17 and 1.23, respectively). In contrast, ‘medium income young’ and ‘low income old’ reported the smallest number of preferred stores per capita (0.5, 0.65, respectively). Based on these values, we conclude that both income and age affect the mix and diversity of preferred stores. The ‘core low income’ population is more inclined to visit multiple stores on a weekly basis, but this number drops rapidly for the elderly.

Table A2 Core agent attribute statistics.

| **Agent Group** | **Weekly trips** | **Motorized trips (%)** | **Stores per respondent** |
| --- | --- | --- | --- |
| CoLwInYg | 3.6 | 59 | 1.17 |
| LwInYg | 3.8 | 65 | 0.88 |
| MeInYg | 8.0 | 67 | 0.5 |
| MeInMiAgOld | 3.7 | 83 | 0.76 |
| CoLwInMiAg | 3.5 | 69 | 1.23 |
| LwInMiAg | 4.5 | 81 | 0.97 |
| CoLwInOld | 0.8 | 85 | 0.94 |
| LwInOld | 2.6 | 89 | 0.65 |

*Observation*.

The outputs collected from the fABM are variables describing each trip (i.e., each trip is recorded separately). These include agent ID, store ID, mode of transportation (motorized/non-motorized), as well as run-specific data: run number, and time step. All output data is used for post-processing analysis.

*Upscaling* (a design concept not included in the ODD protocol).

The agent population is initialized using census counts. The respondent survey, on the other hand, is only a small sample of that population. To assign survey data to agents, an upscaling mechanism is introduced. We follow proportional upscaling with census Monte Carlo sampling (Smajgl et al., 2011). First, individual households from the census estimates are put into predefined age/income groups using a variable "Age of Householder by Household Income in the Past 12 Months" from American Community Survey 5-Year Estimates. Second, survey data is put into the same age/income groups, i.e., respondents are subdivided into Agent Groups based on their reported income and age. Respondent data in a given Agent Group is then used to derive probability distributions, which become distributions of the agent population.

***5. Initialization***

GIS data is loaded into the model as-is (roads, stores, and houses). For stores, their frequency of visits (popularity) is assigned from the empirical data (see the example table in the section that follows). Agents are initialized separately in each agent group. The number of agents in baseline experiments was set to values reported in the table above. Three values are assigned to agent attributes: [1] the number of weekly trips that is sampled from a probability density function (pdf) of trips derived from the surveys (for a given Agent Group), [3] frequency of motorized trips (a proxy of access to a car), and [2] a house in which HHA resides. The house is selected from houses in a given census tract using discrete uniform pdf (i.e., each house in the tract has the same probability of selection). For multi-family houses, the allocation procedure allows for placing multiple HHAs in one building.

HHAs are allocated to houses based on agent group distribution within the census tracts. This allocation was more appropriate than a purely random spread because populations with specific sociodemographic characteristics tend to group in particular locations (socio-spatial segregation).

***6. Input data***

Sampling occurs outside of the model. For sensitivity analysis, quasi-random sampling is employed. Hence, for a given N (number of model executions), all data is generated and placed into an external file that is loaded into the model. The data is then unpacked, and values are assigned to entities (Table A3). Some access restrictions apply to the data underlying the model. The survey data contains confidential information, including income, employment, car ownership, diet, perceived safety, and geocoded addresses, which can be directly tied to individual households.

Table A3 Probability density functions (PDFs) of all trips and motorized trips per Agent Group derived from survey data. Logistic: logistic distribution (parameters: mean and standard deviation), Genextreme: generalized extreme value distribution (parameters: shape, location, and scale), Genlogistic: generalized logistic distribution (parameters: shape, location, scale), Genpareto: generalized Pareto distribution (parameters: shape, location, scale), Gamma: Gamma distribution (parameters: shape, location, scale).

| **Group Name** | **Motorized trips PDF (parameters)** | **All trips PDF (parameters)** |
| --- | --- | --- |
| CoLwInYg | Logistic (2.3415,1.4303) | Genextreme (0.22045, 1.8859, 2.3644) |
| LwInYg | Genextreme (0.21475, 1.2176, 1.6466) | Genlogistic (0.37177, 2.9389, 1.7342) |
| MeInYg | Genextreme (0.59605, 1.6466, 1.892) | Genextreme (0.69787, 1.9163, 2.2241) |
| MeInMiAgOld | Genlogistic (0.24957, 2.7529, 1.2117) | Gamma (1.845, 0, 2.116) |
| CoLwInMiAg | Genlogistic (0.43359, 1.5369, 1.2814) | Genextreme (0.20191, 1.8466, 2.6024) |
| LwInMiAg | Genlogistic (0.55813, 2.1045, s1.575) | Genextreme (0.45444, 1.9705, 2.6648) |
| CoLwInOld | Genextreme (0.08086, 0.35604, 0.61504) | Genpareto (-0.60246, -0.35359, 2.1611) |
| LwInOld | Genpareto (0.12562, -0.52271, 3.1573) | Genlogistic (0.37186, 2.41, 1.5433) |

As an example, we also provide data for stores for one of the Agent Groups:

STOREID FREQENCY CATEGORY

Store2 0.042 Supermarkets

Store6 0.042 Supermarkets

Store13 0.042 Supermarkets

Store14 0.124 Supermarkets

Store17 0.042 Supermarkets

Store20 0.042 Supermarkets

Store24 0.083 Supermarkets

Store26 0.082 Supermarkets

Store36 0.083 Supermarkets

Store41 0.042 Supermarkets

Store49 0.124 Supermarkets

Store55 0.042 Other

Store56 0.042 Other

Store59 0.042 Supermarkets

Store62 0.042 Other

Store63 0.042 Supermarkets

Store64 0.042 Supermarkets

***7. Submodels***

There are no submodels in the fABM.

***8. Supplement***

*Model Development and Structure.*

The model was developed using Python Programming Language (https://www.python.org/) version 3.7 using the Anaconda platform (https://www.anaconda.com/) and Spyder editor. The core model code comprises the main executable script and a package with modules for the household agent, the agent group, the store object, and the house object. Auxiliary code handles input and output. Data is stored in a separate directory. Note that the model does not generate input values on-the-fly, we use an external sample file which stores data for every model run. We performed standard model verification, including debugging, unit tests, test runs, assertions, and extreme combination tests. For validation, we used statistical models that utilized the same data published in LeDoux and Vojnovic (2013) – i.e., we applied model-to-model validation.

*Design of Experiments.*

Sampling for the computational experiments was designed to support variance-based global sensitivity analysis (SA), which decomposes the variance of a distribution of a given output and apportions it to model inputs generating sensitivity indices. For this method of SA, the most efficient sampling is Sobol quasi-random. We established that the minimum number of runs required to converge to acceptable values of the sensitivity indices was 2560. Hence, our final experiment involved 2560 independent model executions, each comprising five-time steps.
